# Editorial: Phosphate handling: from cells to human

**DOI:** 10.3389/fendo.2025.1708453

**Published:** 2025-10-01

**Authors:** Veronica Abate, Ciro Menale, Gianpaolo De Filippo

**Affiliations:** ^1^ Department of Clinical Medicine and Surgery, Federico II University, Naples, Italy; ^2^ Service de médecine interne et rhumatologie, Service de Santé des Armées, Hôpital National d’Instruction des Armées Percy, Clamart, France

**Keywords:** phosphate, bone, FGF23, XLH, parathormon

This Research Topic analyzed phosphate disorders from cellular to human, unveiling the prevalence of its disregarded discrepancies through case reports and clinical trials. Articles reported in this topic investigated phosphate imbalances in several conditions, from pregnancy and children to adults, revealing rare genetic disorders and common side effects to be aware of. Thereby, submissions are of some use both for common clinical practice and for a deeper insight of phosphate balance and of phosphate regulation by hormones, such as 25-hydroxyl vitamin D (25OHD), parathormone (PTH) and fibroblast growth factor 23 (FGF23).

In the first article of this Research Topic, the link between iron infusion and the mineralization process was discussed by Amstad and Burkhardt. In particular, the study focused on iron infusion during the pregnancy and the subsequent consequences on child’s teething, in particular causing dental dysplasia. Observing the similarity in dental dysplasia occurred as a consequence of low serum phosphate in X-linked hypophosphatemia (XLH) and in iron infusion, the role of FGF23 was postulated, probably induced by excess of ferric carboxymaltose administration.

Following the trail of skeletal abnormalities due to phosphate imbalance, a case report by Lu et al. describes a 17-years old boy with hip pain, multiple bone abnormalities (bilateral distal femur, tibiofibular metaphysis, acetabulum, and ilium), low stature, high bone Specific Alkaline Phosphatase and low 25OHD. The genetic test identified a heterozygous inactivating mutation of the ectonucleotide pyrophosphatase/phosphodiesterase 1 (*ENPP1*) gene, responsible for Autosomal-Recessive Hypophosphatemic Rickets 2, that is reported as another FGF23 related form of rickets.

Continuing with the FGF23’s phosphate discrepancy, Zhang et al. report a case of tumor induced osteomalacia. The case demonstrate the effect of FGF23 tumoral overproduction in adult age, and the long path required for the punctual diagnosis. In this case, a 49-year-old woman presented with a history of 2-years pain and weakness, and lasty with femoral neck fracture, before of hospital admission. Biochemical evaluation revealed hypophosphatemia and low renal phosphate reabsorption, providing the diagnosis of osteomalacia. However, FGF23 was not dosed because of the rarity of such an exam. Radiological investigations and later histological examination revealed a phosphaturic mesenchymal tumor. This case underlines the challenging behind phosphate imbalance, not just in children but also in adulthood.

Despite the rarity of these conditions, monoclonal antibodies are available to take out the effect of FGF23 hyperproduction, both in case of acquired adult condition and of inherit childhood’s, namely burosumab. However, little is known about it. An observational study published in the Research Topic by Brener et al. put a light on the cardiovascular effects of the long-term administration of burosumab in children with XLH. In the study, 13 patients have been followed for 2 years after the initial administration of burosumab. As a results, a reduction in cardiovascular risk factors, mostly in the prevalence of both overweight and elevated blood pressure, was demonstrated at the end of the follow-up time. This article demonstrates the safety of burosumab and the variety of effect of FGF23 on human, pointing also to cardiovascular effects of bone hormones, namely the heart-bone axis (1).

Beside FGF23 induced hypophosphatemia, abnormalities in serum levels can also be detected in other cases, as in the study of Improda et al. In this case, a 25OHD deficiency induced a proximal renal tubule dysfunction, named Renal Fanconi Syndrome. The condition is characterized by glucose, phosphate, amino acids and bicarbonates renal loss. The 3-year old Caucasian patient presented with history of epilepsy. He developed valgus deformity of the knees, so that biochemical examination was performed, to reveal low phosphate, low renal reabsorption, low 25OHD and FGF23. Genetic testing showed a pathogenic heterozygous mutation in the solute carrier family 34 member 1 (SLC34A1) gene. The case highlights the need for a differential diagnoses between FGF23 related and not related rickets.

In the last report of XLH, a case report of XLH and tertiary hyperparathyroidism in a 21-year-old man was described by Puliani et al. As first-line recommended therapy, parathyroidectomy was performed. Right after surgery, phosphate and calcitriol was initiated, but the patient developed severe and prolonged hypocalcemia, that needed 2 month to obtain complete correction. The diagnosis of post-surgical hungry bone syndrome was posed. In the years after, the patients required administration of burosumab to reduce the hypersecretion of FGF23. The case unrevealed the complexity of phosphate and calcium balance.

So far, case reports indicate the necessity of an accurate diagnosis of phosphate imbalance, through the study of FGF23, 25OHD and PTH. On the other hand, Perruolo et al. report some laboratory interference, as in the last case report of this Topic. A 27-year-old woman presented with persistently elevated PTH levels, normal serum calcium, phosphate, and vitamin D. In this case, Perruolo et al. performed a polyethylene glycol 6000 precipitation assay to induce a 97% reduction of PTH serum levels. This case indicates that endocrinological diagnostics laboratory must be Critically appraised to correctly identify phosphate imbalances disorders.

It’s also to be considered that calcium-phosphate imbalances can also be caused by interactions with other hormones, as for thyroid hormones. In this last report, Montefusco et al. reports the case of a 61-year-old patient, with history of HIV infection and recent occurrence of weakness, weight loss and panic attacks. The biochemical evaluations revealed hypercalcemia, low PTH and hyperthyroidism, that turned out to be related to Grave’s Disease probably induced by anti-retroviral therapy. On an accurate evaluation, the patient was also diagnosed with a concomitant granulomas due to foreign bodies (silicone injections), that could be also associated to hypercalcemia. Again, a case report indicates the subtle and delicate interactions between bone hormones, to maintain calcium and phosphate balance.

The multiple articles published on this Research Topic underline the complexity of phosphate metabolism and the need of further studies to shed light on the many elements that remain unknown in such complex interaction.
